# Evaluation of Volatile Compounds during the Fermentation Process of Yogurts by *Streptococcus thermophilus* Based on Odor Activity Value and Heat Map Analysis

**DOI:** 10.1155/2020/3242854

**Published:** 2020-07-13

**Authors:** Li Zhang, Si Mi, Ruo-bing Liu, Ya-xin Sang, Xiang-hong Wang

**Affiliations:** Department of Food Science and Technology, Hebei Agricultural University, Baoding 07001, China

## Abstract

The volatile composition of yogurt produced by *Streptococcus thermophilus* fermentation at different time points was investigated by gas chromatography-mass spectrometry combined with simultaneous distillation and extraction. A total of 53 volatile compounds including 11 aldehydes, 10 ketones, 8 acids, 7 benzene derivatives, 13 hydrocarbons, and 4 other compounds were identified in all of the samples. Ketones and hydrocarbons were the predominant volatile components in the early stage, whereas acids were the predominant volatiles in the late stage. The importance of each volatile was evaluated based on odor, threshold, and odor activity values (OAVs). Twenty-nine volatiles were found to be odor-active compounds (OAV > 1), among which (E, E)-2,4-decadienal had the highest OAV (14623–22278). Other aldehydes and ketones such as octanal, dodecanal, 2-nonen-4-one, and 2-undecanone also showed high odor intensity during fermentation. Heat map analysis was employed to evaluate the differences during fermentation. The results demonstrated that the volatile profile based on the content and OAVs of volatile compounds enables the good differentiation of yogurt during fermentation.

## 1. Introduction

Yogurt is a very popular fermented milk product that is widely consumed fermented milk product worldwide. Its unique sensory, odor, and texture qualities are formed during the process of microbial fermentation [1]. Of them, odor is one of the most important factors that determines the acceptability and preference of yogurts. More than 100 volatiles including carbonyl compounds, alcohols, acids, esters, hydrocarbons, aromatic compounds, sulfur-containing compounds, and heterocyclic compounds were detected in yogurt at low to trace concentrations [2–4].

Yogurt is usually produced using a mixture of homofermentative lactic acid bacteria such as *Streptococcus thermophilus* and *Lactobacillus bulgaricus* as the starter culture [5, 6]. A large number of studies have been conducted on the factors that affect the flavor of yogurt such as the source of milk [7–9], milk components [6, 10], starter cultures [11], process technology [12, 13], additives [14], and storage conditions [9, 11]. Among them, the starter cultures play a key role in the formation of flavor compounds. The difference in aroma has been attributed to the presence of different bacteria, so an increasing amount of attention has been paid to the influence of single bacteria on the flavor of yogurt. Imhof et al. (1995) reported that volatile organic compounds are produced by *Thermophilic lactobacilli* and *Mesophilic lactococci* single strain dairy starter and considered 2,3-butanedione, 2,3-pentanedione, dimethyl sulfide, and benzaldehyde to be differential compounds formed by different strains [15]. Wang et al. (2015) isolated the *L. lactis* strain from Tibetan kefir grains, which was used for yogurt making, and 25 types of flavor compounds including acetaldehyde, diacetyl, and 2-heptanone were generated [16].

*S. thermophilus* is an important bacterial strain used for the industrial production of yogurt, and it has an important influence on the odor quality of yogurt. Fermentation is an important step in yogurt processing and is a crucial step for the formation of yogurt odor quality. Hence, the formation and change in volatile components when yogurt is fermented by *S. thermophiles* are also of great significance to the quality formation and processing control of yogurt [11]. However, few studies have focused on this question. In addition, it is worth paying attention to which volatile components contribute to odor quality during yogurt fermentation. The odor activity value (OAV) is an important method used to select key odor components. It has been widely introduced to choose impact odorants in dairy product such as milk [17, 18], milk power [19, 20], and cheese [21–23]. Tian et al. (2019) reported that odor characteristic components in yogurt are generated by *L. plantarum* fermentation using OAV and confirmed that diacetyl, 2-heptaone, and 2-nonanone are important odor components [24].

The objective of this study was to monitor the change of volatile compounds during the fermentation process of yogurts by *S. thermophilus*. To this end, simultaneous distillation and extraction (SDE) and gas chromatography-mass spectrometry (GC-MS) were utilized. The odor activity value of the identified volatiles was calculated to evaluate the contributions of compounds on the overall aroma of the investigated samples. The present work could facilitate a better understanding of volatile composition in a typical yogurt and can be expected to be used for the improvement of the yogurt quality.

## 2. Materials and Methods

### 2.1. Yogurt Preparation

Pasteurized full-fat milk obtained from a local factory (Hebei Sanyuan Food Co., Ltd, Hebei, China) was fortified with 6.0% sugar. Homogenization was performed at 5 pa pressure and heat treatment at 98°C for 15 min. Then milk was immediately cooled to about 41°C in a water bath and inoculated with a starter culture of *S. thermophilus* S2 (Hebei Inatural Biotech Co., Ltd., Hebei, China). The prepared solution (1000 mL) was added to sterile pots, which then were hermetically sealed after which the solution was fermented at 41°C for 6 h (until pH 4.2 was reached). Samples were taken at 0, 1.5, 3.0, 4.5, and 6.0 h during fermentation. The collected yogurts were stored at −24°C until subsequent analysis.

### 2.2. Extraction of Volatile Compounds

The volatile compounds of yogurt were extracted using simultaneous distillation and extraction (SDE) in a Likens-Nickerson apparatus. A mixture of 200 g sample and 2 L deionized water was placed in a 5 L flask and connected to a Likens-Nickerson apparatus. Aqueous 2,4,6-trimethyl-pyridine (40 mg·kg^−1^) was added as an internal standard. Then a U-tube apparatus was filled with water. An additional 50 mL diethyl ether was added. An additional 10 mL diethyl ether was poured into a finger-shaped bottom flask. Both flasks (containing diethyl ether and yogurt mixture, respectively) were heated to boiling. The extraction time was 2 h. After extraction, the distillate in the 100 mL flask was dried over anhydrous sodium sulfate (5 g), concentrated using vacuum rotary evaporation, and then stored in headspace vials. Extraction of each sample was performed in triplicate.

### 2.3. GC-MS Analysis

The analysis of volatile compounds was conducted using the Agilent 6890 GC System equipped with the Agilent 5975 MS (Agilent Technologies, Santa Clara, CA, USA) and fitted with the DB-5 capillary column (30 m × 0.25 mm ID, 0.25 *μ*m film thickness; J&W Scientific, Folsom, CA, USA). Ultrahigh purity helium (≥99.999%) was employed as the carrier gas with a constant flow rate of 1 mL·min^−1^, and 1 *μ*l extraction of sample was injected at 250°C in splitless mode. The oven temperature was programmed at 40°C for 2 min and then ramped to 220°C at a rate of 6 °C·min^−1^ and held at 220°C for 5 min. The mass spectrometer was operated in the electron impact ionization mode at a voltage of 70 eV and ion source temperature at 230°C. Mass spectra were taken over an m/*z* range of 30–400. Retention indices were calculated after analyzing C_8_–C_20_*n*-alkane series under the same chromatographic conditions.

### 2.4. Identification of Volatile Compounds

The volatile components were identified by comparing their mass spectra to mass spectra from mass spectral libraries (NIST 05, WILEY 7.0) and to previously published data. These volatiles were further confirmed by matching their linear retention indices (LRIs) and odor descriptions in the literature [3, 24, 25]. The LRIs were computed according to the following equation [26]:

(1) $$\begin{array}{c} \text{LRI}=100\times \left(\frac{Rt\;\left(i\right)-Rt\;\left(n\right)}{Rt\;\left(n+1\right)-Rt\;\left(n\right)+n}\right), \end{array}$$

where *Rt* (i) is the retention time of the individual compound under investigation (i) and *Rt* (*n*) and *Rt* (*n*+1) refer to the retention times of *n*-alkanes (C_8_–C_22_; Supelco Analytical, Sigma, St. Louis, MO, USA) that elute before and after the target compound (i) for the same chromatographic conditions.

Aqueous 2, 4, 6-trimethylpyridine (40 mg·kg^−1^) was used as internal standard for quantitative analysis. The quantitative calculation (mg·kg^−1^) was based on

(2) $$\begin{array}{c} \mathrm{concentration}\;\left.\left(\text{each compound}\right)\right)=\frac{\text{concentration}\;\left(\text{internal standard}\right)\times \text{peak area}\left(\text{each compound}\right)}{\text{peak area}\left(\text{internal standard}\right)}. \end{array}$$

OAV for each volatile compound was calculated using the equation OAV = *c*/*t*, where *c* is the total concentration of the compound in the yogurt and *t* is the odor threshold value. Compounds with OAV >1 were considered as odor-active compounds [27].

### 2.5. Statistical Analyses

All volatile concentration data represent the average of the triplicate measurements. Analysis of variance (ANOVA) followed by Turkey and Dunnett's multiple comparison test was adopted to evaluate the differences in volatile concentrations among yogurt samples. Heat map was performed using MultiExp to assess differences in volatile compounds and odor-active compounds, respectively. Unless specified, *p* values of <0.05 were considered statistically significant.

## 3. Results and Discussion

### 3.1. Volatile Composition of Yogurts by *S. thermophilus* at Different Fermentation Times

The concentrations of identified volatiles in the yogurts are provided in Table 1. A total of 53 volatile compounds including 11 aldehydes, 10 ketones, 8 acids, 7 benzene derivatives, 13 hydrocarbons, and 4 other compounds were detected. It can be seen from Table 1 that fermentation time had a significant impact on the volatile profiles of yogurts. A total of 35, 38, 41, 40, and 36 volatile compounds, for which the total concentrations ranged from 1042.44 to 2810.78 mg·kg^−1^, were detected in the fermented samples collected at 0, 1.5, 3, 4.5, and 6.0 h, respectively. Twenty-three constituents were simultaneously present in all of the samples. Ketones (42.20%) and hydrocarbons (54.45%) were the predominant volatile components in 0 h sample, whereas acids (64.41%) were the main constituents in 6.0 h sample. In addition, ketones (11.28%) and hydrocarbons (19.11%) also showed relative low content in 6.0 h sample.

A total of 11 aldehyde compounds were detected, being 8, 9, 9, 8, and 7 for the yogurts collected at 0, 1.5, 3, 4.5, and 6.0 h, respectively. Of these aldehyde compounds, three of them contained benzene, while the other eight aldehydes belonged to *n*-alkanals. The concentration of aldehydes was relatively low, and the total concentrations of aldehydes, ranging from 36.64 to 17.99 mg·kg^−1^, were significantly different (*p* < 0.05) in samples fermented at different times. Of the 11 detected aldehydes, benzaldehyde, octanal, nonanal, 2,4-dimethyl-benzaldehyde, (E,E)-2,4-decadienal, and tetradecanal were detected in all samples during the whole fermentation process and presented obvious dynamic changes. Only benzeneacetaldehyde and (E)-2-decenal were detected in the intermediate stage (3.0 h). Only undecanal, and not decanal and dodecanal, was present in the end of fermentation (6.0 h). Aldehydes usually dynamically change in yogurt during fermentation. They can be derived from the catabolism of amino acids [28] or the decomposition of hydroperoxide, which is an oxidation product of unsaturated fatty acids [2]. At the same time, aldehydes can be reduced to alcohols by alcohol dehydrogenases or oxidized to carboxylic acids by an aldehyde dehydrogenase [29]. Therefore, changes in the content of aldehydes have an important influence on the composition of volatile components during the fermentation of yogurt by *S. thermophiles*.

Ten ketones were detected in all samples, of which seven belonged to methyl ketones. The total concentrations of ketones were between 267.55 (3.0 h) and 562.43 mg·kg^−1^ (4.5 h). Six similar ketone compounds, including 2-heptanone, 2-nonanone, 2-undecanone, 2-tridecanone, 2-pentadecanone, and 6-heptyltetrahydro-2H-pyran-2-one, were detected in all samples. 2-Nonen-4-one and (Z)-dihydro-5-(2-octenyl)-2(3H)-furanone were lacking in 0–3.0 h and 0–1.5 h fermented yogurt samples, and 2-decanone and 2-dodecanone were lacking in 4.5 h and 6.0 h fermented yogurt samples. Ketones were the dominant volatile components in the initial stage of fermentation, and 2-heptanone, 2-nonanone, 2-undecanone 2-tridecanone, and (Z)-dihydro-5-(2-octenyl)-2(3H)-furanone exceeded 5% in 0, 1.5, and 3.0 h yogurt samples. There was little change in content of 2-undecanone and 2-tridecanone during the initial stage (0–1.5 h); their content peaked in the intermediate stage (3.0 h) and dropped sharply at 4.5 h.

All of the values were expressed as mean (*n* = 3) ± standard deviation; RI : retention index; SD : standard deviation; nd : not detected; mean values in the same row with different letters indicate the significant differences between clusters (*p* < 0.05) were maintained at relatively low levels in the late stage (4.5–6.0 h). Ketones are common volatile compounds in dairy products, and raw milk is an important source of ketones. Fermentation by *S. thermophiles* has an important effect on the content of ketones. At first, the preheating treatment accelerates the formation of ketone compounds in the milk [20]. Then ketone compounds can be generated from autoxidation reactions of unsaturated fatty acids during fermentation [20]. For example, 2-heptanone is formed by *β*-oxidation of saturated fatty acids followed by decarboxylation or by decarboxylation of *β*-ketoacids naturally present in milk fat [30].

Eight acids were detected in all yogurt samples. Acids were the group of compounds most affected by *S. thermophilus* fermentation. The content of acids significantly increased (*p* < 0.05) during the whole fermentation process. In the initial stage (0 h) of yogurt fermentation, only dodecanoic acid and tetradecanoic acid with a total content 11.90 mg·kg^−1^ were detected. At the end of fermentation (6.0 h), eight acids with 1866.56 mg·kg^−1^ total content were detected, and acids became the predominant volatile components in sample. A large number of new acid compounds were present in yogurt at different fermentation times. For example, octanoic acid, decanoic acid, hexanoic acid, undecanoic acid, nonanoic acid, and tridecanoic acid were first detected in 1.5, 3.0, 4.5, and 6.0 h yogurt samples, and the content of these compounds increased along with the increasing of fermentation time. All of the acids belonged to straight chain carboxylic acids in fermented yogurt sample. Lipolysis is thought to be the main pathway for the formation of carboxylic acids, followed by lactose metabolism [31]. Carboxylic acids not only are aroma components but also can serve as precursors for the formation of methyl ketones, alcohols, lactones, aldehydes, and esters [31]. Among the various detected compounds, the change in acid compounds was the largest not only in composition of the component but also in the content of the component. These acids may have an important impact on the final odor quality of the yogurt.

Seven benzene derivatives were detected in yogurts during fermentation by *S. thermophiles*. These compounds were also less abundant in samples. The total concentrations of benzene derivatives were about 30 mg·kg^−1^ and were significantly different (*p* < 0.05) in samples which were fermented under different fermentation time. With the exception of 1-methyl-naphthalene and 2,3-dimethyl-naphthalene, which were not detected in the final stage fermentation sample, other benzene derivatives including ethylbenzene, p-xylene, styrene, 1,2,4,5-tetramethyl-benzene, and naphthalene were present in all samples. Throughout the fermentation process, benzene derivatives showed relatively high stability. They are usually from milk of cows fed on pasture grass [32] and maintained a relatively stable content during the entire fermentation process.

Hydrocarbons were the richest group in compounds identified in all of the samples, and 13 hydrocarbons were detected in yogurts during fermentation by *S. thermophiles*. Seven, eight, nine, nine, and six hydrocarbons were detected from 0 to 6.0 h fermentation samples, respectively. The total content of hydrocarbons did not significantly change (*p* > 0.05) during the fermentation process. Nonane, which was the dominant hydrocarbon of about 550 mg·kg^−1^, kept a relatively stable content during the whole fermentation process. The amounts of other hydrocarbons were generally low and fluctuated during fermentation. 4,8-Dimethyl-undecane and pentadecane were only present in the initial stage (0 h). Decane and tetradecane were present in the early stage (0–4.5 h), 1-dodecene and 1-tetradecene were present in the middle stage (1.5–4.5 h), undecane, tridecane, 1-pentadecene, and 1-heptadecene were mainly present in yogurts fermented after 1.5 h, and nonane and hexadecane were the most frequently detected hydrocarbons during the entire stage (0–6.0 h). Hydrocarbons are generally derived from lipid oxidation [31]. Despite the frequent detection of hydrocarbons in all samples, they do not contribute to yogurt aroma due to their low detection amounts and high perception threshold [33].

In addition, there were four other compounds detected in the yogurts during the fermentation process including diethyl disulfide, 2-pentyl-furan, limonene, and 2,4-bis(1,1-dimethylethyl)-phenol. Most of them were common volatile compounds in yogurt. Diethyl disulfide, which is usually produced by microbial metabolism of sulfur-containing amino acids or decomposition of sulfydryl groups during heat treatment before yogurt fermentation [34], was detected in the middle fermentation stage. 2-Pentyl-furan, a secondary oxidation product of linoleic acids, was only present in the 0 h sample [35]. Limonene may be from forage, which is transferred to milk from a cow's feed [32]. There was no significant change in the early stage, and the decrease in the later period was obvious. 2,4-Bis(1,1-dimethylethyl)-phenol significantly decreased in the earlier fermentation period.

### 3.2. OAVs of Volatile Compounds in Yogurts by *S. thermophilus* at Different Times

Although dozens of volatile compounds were detected in yogurt after *S. thermophilus* fermentation at different time points, not all of the components had a large impact on the overall aroma characteristic of the samples. To evaluate the contribution of various volatile compounds on olfactory impression, characteristic aroma compounds were determined by OAVs, which were calculated on the basis of the ratio of the concentration of the compound to odor threshold. The aroma characteristics of 30 volatile compounds were described in previous works [2, 24, 25]. As shown in Table 2, OAVs were >1 in 29 volatile compounds. These compounds played a critical role in yogurt by *S. thermophilus* fermentation at different time points. A total of 19, 19, 20, 22, and 20 odor-active compounds were detected in yogurt at 0, 1.5, 3.0, 4.5, and 6.0 h fermentation, respectively.

As shown in Table 2, aldehydes made up the largest group of odor-active volatiles, of which nine aldehydes were detected as odor-active compounds in the samples. Due to lower thresholds, even lower levels of aldehydes were detected, which had important effects on the odor quality of the yogurt samples. Nonanal, (E,E)-2,4-decadienal, and dodecanal showed extremely high OAVs (>1000) and were responsible for the green, woody odor, so they made great contributions to the flavor of yogurt during fermentation by *S. thermophiles*. It is worth noting that the OAV of (E,E)-2,4-decadienal was between 14,623 and 22,278 and was the most important odor-active compound in the yogurt samples. Due to the extremely low odor threshold of (E,E)-2,4-decadienal (0.0001 mg·kg^−1^, fatty and green), trace changes had a huge impact on odor intensity. Benzeneacetaldehyde (sweet, flora), decanal (fatty), and undecanal (fatty) had high OAVs (100–1000) and were only detected in 3.0, 1.5, and 6.0 h yogurt samples, respectively. Nonanal, with a “sweet, floral, citrus, and grass” odor, had high OAVs (100–1000) in the initial phase and medium OAVs (10–100) in the last phase. Except for the late stage, decanal had medium OAVs in the yogurt samples. Benzaldehyde, which contributed almond and burnt sugar, had low OAVs (1–10) throughout the fermentation process. Among the detected odor-active aldehydes, nonanal and decanal were also detected in yogurt fermented by *L. plantarum*, but they did not contribute odor to sample due to OAV <1 [24]. Nonanal, (E)-decenal, and (E,E)-2,4-decadienal reportedly contribute “fatty,” “fruity,” and “fruity,” odors, respectively, to fermented camel milk [36]. In addition, octanal and decanal contribute “fatty, lemon, green” and “green” odors to dairy products such as cheese [37].

Six ketones were found to affect the aroma characteristic of yogurt by *S. thermophiles* at different time points. Among them, 2-none-4-one, which contributed a “fruity, floral” odor, showed extremely high OAVs (>1000) in 4.5 h (3391) and 6.0 h (2870) samples due to the low threshold (0.0009 mg·kg^−1^). 2-Undecanone contributed an “orange, grass, fresh” odor with extremely high OAVs in 0–3.0 h samples (1102–1427) and high OAVs in 4.5–6.0 h yogurt samples (482–572). 2-Heptaone and 2-nonaone provided “fresh cream” and “grass, fruity, floral” odors with high OAVs during the whole fermentation process. 2-dodecanone contributed an “orange, grass, fresh” odor in the 0, 1.5, and 3.0 h samples with 24, 28, and 34 OAVs that belonged to medium OAVs. Among the odor-active ketones, 2-heptone, 2-nonanone, and 2-undecanone contributed “fresh cream,” “grass, fruity, floral,” and “foam grass” odors to yogurt fermented by *L. plantarum* with medium OAVs [24]. 2-Decanone, which contributes a “fresh cream” odor, was identified as an odor-active compound in fermented camel milk [36].

Among the acids with odor descriptions, most had OAVs >1 except for tridecanoic acid and mainly contributed odor in the later stage of fermentation. In the 4.5 h sample, hexanoic acid, undecanoic acid, dodecanoic acid, and tetradecanoic acid provided a “rancid” odor with low OAVs, and octanoic acid and decanoic acid also provided a “rancid” odor with medium OAVs. In the 6.0 h sample, the OAVs of hexanoic acid, octanoic acid, undecanoic acid, dodecanoic acid, and tetradecanoic acid increased to 10–100 and had medium odor intensity, whereas the OAV of decanoic acid increased to 260 and had high odor intensity. Hexanoic acid and octanoic acid with characteristic of “spicy, rancidity” and “chess, sweaty” odors were detected as odor-active compounds in yogurt fermented by *L. plantarum* with medium OAVs [24]. Undecanoic acid, decanoic acid, dodecanoic acid, and tetradecanoic acid provide odors such as “fatty,” “sweet,” “floral,” and “waxy” in dairy products such as fermented camel milk [36] and cheese [37].

Five odor-active benzene derivatives including ethylbenzene, p-xylene, styrene, naphthalene, and 1-methyl-naphthalene give a “phenolic” odor to samples. Ethylbenzene and p-xylene had medium odor intensities, and styrene, naphthalene, and 1-methyl-naphthalene had high odor intensities. Most of them retained a relatively stable odor intensity throughout the fermentation process. Benzene derivatives such as ethylbenzene and p-xylene reportedly provide “phenol, spice” and “leathery” aromas to yogurt. Other compounds were detected as active odor compounds in the samples. Diethyl disulfide and 2-pentyl-furan had high OAVs at different fermentation times by *S. thermophiles*. Limonene had medium OAVs in yogurt samples fermented in 0–3.0 h and had low OAVs in yogurt samples fermented in 4.5–6.0 h.

### 3.3. Heat Map Analysis of Volatile Compounds in Yogurts during Fermentation

**Figure 1:**
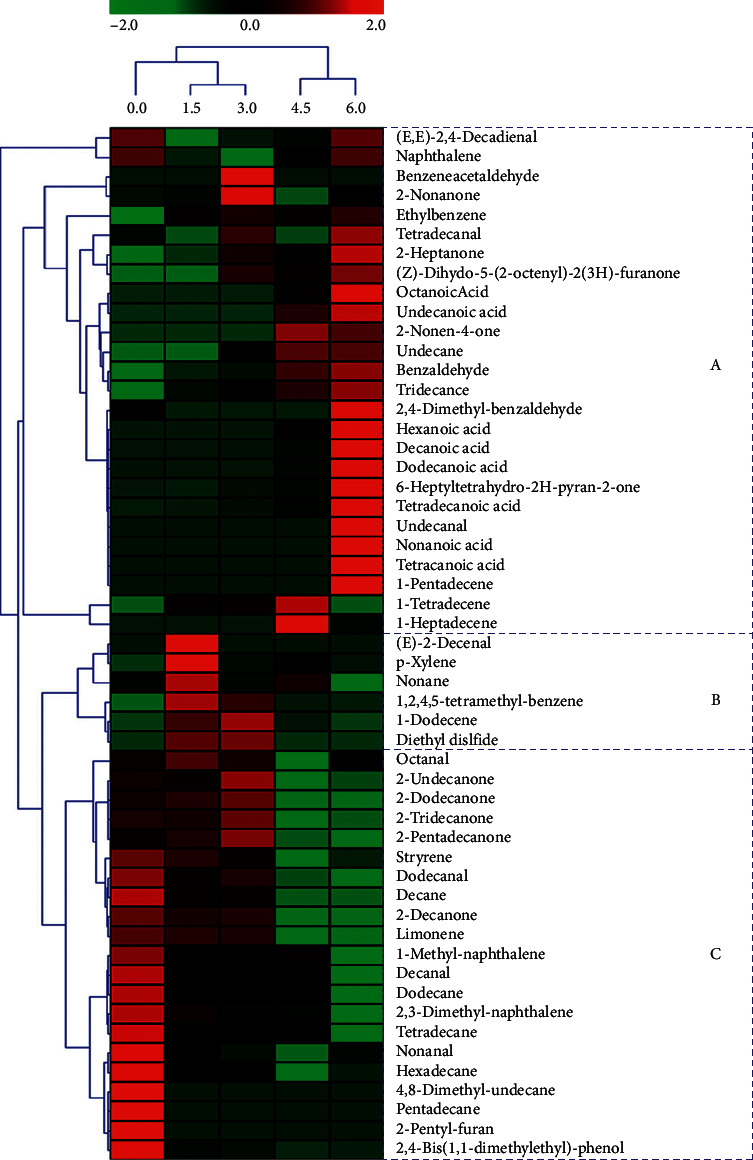
Heat map plots clusters of volatile compounds in yogurt fermented by *S. thermophilus* at different time points based on the content. Green colors indicate that volatile compound levels were less than the mean levels during yogurt fermentation, while red colors indicate that volatile compound levels were higher than the mean levels.

A heat map was generated to show the variation in content of volatile compounds in yogurt during fermentation by *S. thermophiles* (Figure 1). Green colors indicate that the volatile compound levels were less than the mean level during yogurt fermentation, whereas the red colors indicate that volatile compound levels were higher than the mean levels. The samples collected from different stages were clustered into three clusters: the first cluster contained the 0 h sample, the second cluster contained the 1.5 and 3.0 h samples, and the third cluster contained the 4.5 and 6.0 h samples. According to the content of the volatile compounds, the samples were divided into three groups: A, B, and C. A group included six acids (octanoic acid, undecanoic acid, hexanoic acid, decanoic acid, dodecanoic acid, and tridecanoic acid), six aldehydes ((E,E)-2,4-decadienal, benzeneacetaldehyde, tetradecanal, benzaldehyde, 2,4-dimethyl-benzaldehyde, and undecanal), five ketones (2-nonanone, 2-heptanone, (Z)-dihydro-5-(2-octenyl)-2(3H)-furanone, 2-nonen-4-one, and 6-heptyltetrahydro-2H- pyran-2-one), five hydrocarbons (undecane, tridecane, 1-pentadecene, 1-tetradecene, and 1-heptadecene), and two benzene derivatives (naphthalene and ethylbenzene). Most of the acids and unsaturated hydrocarbons were focused in this group. These compounds usually showed a high content in the later stage. The B group included six compounds, namely, (E)-2-decenal, p-xylene, nonane, 1,2,4,5-tetramethyl- benzene, 1-dodecene, and diethyl disulfide, which showed a high content in the medium stage. The C group, which had a high content in the original stage, included four aldehydes (octanal, dodecanal, decanal, and nonanal), four ketones (2-undecanone, 2-dodecanone, 2-tridecanone, and 2-petadecanone), five hydrocarbons (decane, dodecane, tetradecane, hexadecane, and pentadecane), three benzene derivatives (styrene, 1-methyl-naphthalene, and 2,3-dimethyl-naphthalene), and three other compounds (limonene, 2-pentyl-furan, and 2,4-dis(1,1-dimethylethy)-phenol). Conclusions section should clearly explain the main findings and implications of the work, highlighting its importance and relevance.

**Figure 2:**
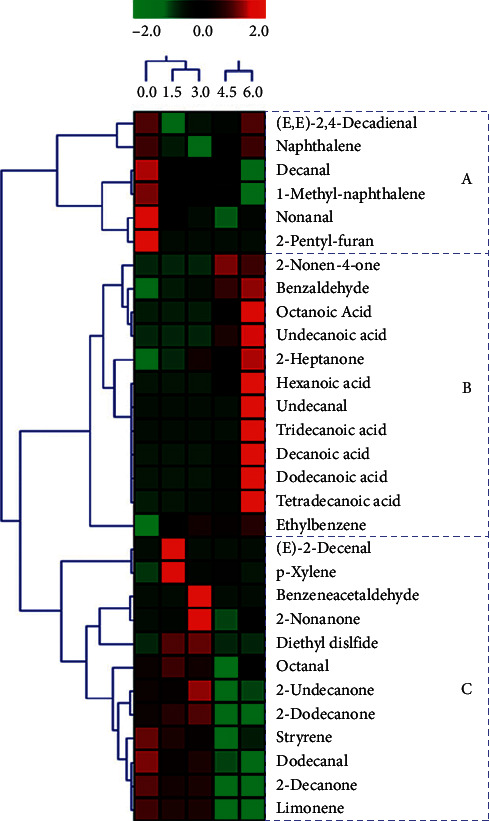
Heat map plots clusters of volatile compounds in yogurt fermented by *S. thermophilus* at different time points based on the OAVs. Green colors indicate that volatile compound levels were less than the mean levels during yogurt fermentation, while red colors indicate that volatile compound levels were higher than the mean levels.

Figure 2 shows the heat map of variation in the OAVs of volatile compounds in yogurt during fermentation. There were similar variations in OAVs with the content of volatile compounds from different fermentation times. Yogurt fermented for 0 h belonged to the first cluster, yogurts fermented for 1.5 and 3.0 h belonged to the second cluster, and yogurt fermented 4.5 and 6.0 h belonged to the third cluster. According to the OAVs that presented the odor intensity of the volatile compounds, the samples were divided into A, B, and C groups. Group A contained six compounds, which showed a high odor intensity in the 0 h yogurt sample including three aldehydes ((E,E)-2,4-decadienal, decanal, nonanal), two benzene derivatives (naphthalene, 1-methyl-naphalene), and 2-pentyl-furan. (E, E)-2,4-decadienal had the highest OAVs in most of yogurts located in this group. The B group contained 12 compounds, which showed high odor intensity in the end stage of fermentation including 7 acids (octanoic acid, undecanoic acid, hexanoic acid, tridecanoic acid, decanoic acid, dodecanoic acid, and tetradecanoic acid), 2 aldehydes (benzaldehyde and undecanal), 2 ketones (2-nonen-4-one, and 2-heptanone), and 1 benzene derivative (ethylbenzene). In this group, “rancid” was the important odor characteristic. The C group contained 12 compounds, which showed a high odor intensity in the 0–4.5 h yogurt samples including 4 aldehydes ((E)-2-decenal, benzeneacetaldehyde, octanal, and dodecanal), 4 ketones (2-nonanone, 2-undecanone, 2-dodecanone, and 2-decanone), 2 benzene derivatives (p-xylene and styrene), and 2 other compounds (diethyl disulfide and limonene). Octanal, dodecanal, and 2-undecanone had OAVs exceeding 1000, which had great influence on this group.

## 4. Conclusions

The volatile component profiles of yogurts had significant changes during the fermentation process by *S. thermophilus*. Ketones and hydrocarbons were the predominant volatile components at the early stage, with the extension of fermentation time acids becoming the predominant volatiles. “Fatty, green” provided by (E,E)-2,4-decadienal was the dominant odor in the whole yogurts fermentation process, and “rancid” odor intensity increased during fermentation. The heat map analyses could clearly differentiate the volatile compounds and OAVs of yogurts by *S. thermophilu*s fermentation at different time points.

## Figures and Tables

**Table 1:** Content of volatile compounds in yogurt by *S. thermophilus* at different fermentation times.

RI	Compound names	Content (mg·kg−1)				
		0 h	1.5 h	3.0 h	4.5 h	6.0 h
	*Aldehydes*					
955	Benzaldehyde	1.34 ± 0.18^d^	1.86 ± 0.31^c^	2.03 ± 0.17^c^	3.23 ± 0.08^b^	3.68 ± 0.26^a^
1004	Octanal	1.45 ± 0.13^a^	1.65 ± 0.39^a^	1.49 ± 0.08^a^	0.76 ± 0.26^b^	1.28 ± 0.24^a^
1045	Benzeneacetaldehyde	nd	nd	1.26 ± 0.28^a^	nd	nd
1106	Nonanal	8.54 ± 2.01^a^	4.28 ± 0.61^b^	3.34 ± 0.31b^c^	1.68 ± 0.24^c^	3.48 ± 0.12^bc^
1206	Decanal	2.35 ± 0.07^a^	1.20 ± 0.20^b^	1.14 ± 0.13^b^	1.28 ± 0.18^b^	nd
1216	2,4-Dimethyl-benzaldehyde	6.62 ± 0.34^b^	3.41 ± 0.31^c^	3.56 ± 0.21^c^	3.54 ± 0.54^c^	16.49 ± 2.14^a^
1263	(E)-2-Decenal	nd	0.64 ± 0.06^a^	nd	nd	nd
1308	Undecanal	nd	nd	nd	nd	2.56 ± 0.20^a^
1318	(E,E)-2,4-Decadienal	2.22 ± 0.28^a^	1.46 ± 0.16^b^	1.71 ± 0.12^b^	1.78 ± 0.06^b^	2.23 ± 0.14^a^
1410	Dodecanal	8.75 ± 1.14^a^	5.30 ± 0.55^b^	6.29 ± 0.79^b^	0.92 ± 0.05^c^	nd
1614	Tetradecanal	5.38 ± 0.98^bc^	4.74 ± 0.37^c^	6.34 ± 0.66^ab^	4.80 ± 0.39^c^	6.83 ± 0.98^a^
	Subtotal	36.64 ± 4.25^a^	24.54 ± 2.15^b^	27.15 ± 2.11^b^	17.99 ± 0.45^c^	36.54 ± 2.66^a^
	*Ketones*					
891	2-Heptanone	54.12 ± 2.90^c^	56.57 ± 7.84^c^	65.25 ± 6.59^ab^	61.17 ± 4.63^ab^	71.84 ± 7.59^a^
1094	2-Nonanone	64.67 ± 4.98^b^	65.34 ± 6.84^b^	79.52 ± 3.98^a^	60.84 ± 4.12^b^	66.71 ± 6.37^b^
1130	2-Nonen-4-one	nd	nd	nd	3.05 ± 0.57^a^	2.58 ± 0.23^a^
1199	2-Decanone	4.52 ± 0.40^a^	3.38 ± 0.29^b^	3.54 ± 0.35^b^	nd	nd
1296	2-Undecanone	88.21 ± 10.22^b^	84.38 ± 7.37^b^	114.18 ± 12.05^a^	38.61 ± 1.16^c^	45.79 ± 4.94^c^
1399	2-Dodecanone	1.98 ± 0.36^b^	2.26 ± 0.37^b^	2.78 ± 0.36^a^	nd	nd
1498	2-Tridecanone	112.01 ± 14.09^b^	108.92 ± 8.41^b^	135.40 ± 14.91^a^	35.88 ± 2.23^c^	45.10 ± 2.02^c^
1658	(Z)-Dihydro-5-(2-octeny)-2(3H)-furanone	nd	nd	7.64 ± 0.49^b^	6.13 ± 0.68^c^	10.33 ± 0.23a
1699	2-Pentadecanone	79.83 ± 8.94^b^	90.08 ± 9.04^b^	118.29 ± 10.71^a^	24.67 ± 2.19^c^	17.51 ± 2.69^c^
1710	6-Heptyltetrahydro-2H-pyran-2-one	34.58 ± 1.90^b^	34.26 ± 2.43^b^	35.83 ± 3.35^b^	37.20 ± 1.25^b^	57.08 ± 0.92^a^
	Subtotal	439.92 ± 33.78^b^	445.19 ± 41.72^b^	562.43 ± 35.72^a^	267.55 ± 12.98^c^	316.94 ± 24.50^c^
	*Acids*					
1008	Hexanoic acid	nd	nd	nd	5.17 ± 0.25^b^	30.26 ± 3.54^a^
1194	Octanoic acid	nd	0.64 ± 0.07^c^	0.81 ± 0.07^c^	55.85 ± 5.61^b^	149.38 ± 11.51^a^
1285	Nonanoic acid	nd	nd	nd	nd	18.36 ± 1.83^a^
1377	Decanoic acid	nd	nd	2.68 ± 0.28^b^	70.75 ± 4.47^b^	782.24 ± 78.33^a^
1473	Undecanoic acid	nd	nd	nd	9.88 ± .49^b^	15.80 ± 0.40^a^
1568	Dodecanoic acid	10.92 ± 2.43^c^	2.21 ± 0.17^c^	6.18 ± 0.79^c^	66.71 ± 7.11^b^	564.28 ± 58.05^a^
1666	Tridecanoic acid	nd	nd	nd	nd	5.97 ± 0.78^a^
1765	Tetradecanoic acid	0.98 ± 0.00^c^	5.26 ± 0.42^c^	20.96 ± 3.87b^c^	41.66 ± 3.07^b^	300.27 ± 32.23^a^
	Subtotal	11.90 ± 2.43^c^	8.11 ± 0.29^c^	30.64 ± 4.96^c^	250.03 ± 20.26^b^	1866.56 ± 174.63^a^
	*Benzene derivatives*					
852	Ethylbenzene	3.98 ± 0.41^b^	5.25 ± 0.52^a^	5.44 ± 0.28^a^	5.28 ± 0.97^a^	5.54 ± 0.21^a^
860	p-Xylene	13.19 ± 1.68^a^	15.12 ± 0.88^a^	13.52 ± 0.49^a^	13.63 ± 1.88^a^	13.42 ± 0.83^a^
884	Styrene	12.71 ± 1.97^a^	12.27 ± 1.81^a^	11.93 ± 1.25^a^	10.19 ± 0.74^a^	11.05 ± 0.84^a^
1118	1,2,4,5-Tetramethyl-benzene	0.99 ± 0.15^b^	2.33 ± 0.11^a^	1.98 ± 0.34^a^	1.26 ± 0.06^b^	1.22 ± 0.18^b^
1183	Naphthalene	1.14 ± 0.07^a^	0.86 ± 0.08^a^	0.71 ± 0.10^c^	1.00 ± 0.08^ab^	1.14 ± 0.05^a^
1289	1-Methyl-naphthalene	3.49 ± 0.15^a^	2.00 ± 0.18^b^	2.09 ± 0.16^b^	2.26 ± 0.18^b^	nd
1417	2,3-Dimethyl-naphthalene	1.05 ± 0.04^a^	0.65 ± 0.09^b^	0.44 ± 0.03^c^	0.43 ± 0.08^c^	nd
	Subtotal	36.56 ± 3.83^ab^	38.48 ± 2.97^a^	36.12 ± 2.04^ab^	34.05 ± 2.37^ab^	32.36 ± 1.95^b^
	*Hydrocarbons*					
900	Nonane	539.67 ± 72.04^a^	564.57 ± 46.61^a^	538.43 ± 58.91^a^	550.29 ± 46.52^a^	522.47 ± 27.75^a^
1000	Decane	1.11 ± 0.15^a^	0.58 ± 0.07^b^	0.60 ± 0.04^b^	nd	nd
1100	Undecane	nd	nd	0.74 ± 0.10^b^	1.35 ± 0.09^a^	1.34 ± 0.15^a^
1197	1-Dodecene	nd	3.39 ± 0.30^b^	4.35 ± 0.61^a^	0.67 ± 0.13^c^	nd
1200	Dodecane	1.35 ± 0.10^a^	0.66 ± 0.10^b^	0.76 ± 0.07^b^	0.66 ± 0.03^b^	nd
1300	Tridecane	nd	0.61 ± 0.08^c^	0.72 ± 0.09^c^	1.21 ± 0.15^b^	1.61 ± 0.16^a^
1224	4,8-Dimethyl-undecane	1.09 ± 0.16^a^	nd	nd	nd	nd
1396	1-Tetradecene	nd	1.04 ± 0.10^b^	1.09 ± 0.28^b^	2.04 ± 0.10^a^	nd
1400	Tetradecane	3.45 ± 0.26^a^	1.74 ± 0.20^b^	1.49 ± 0.15^b^	1.38 ± 0.26^b^	nd
1494	1-Pentadecene	nd	nd	nd	nd	6.41 ± 0.48^a^
1500	Pentadecane	8.24 ± 0.61^a^	nd	nd	nd	nd
1600	Hexadecane	12.68 ± 0.87^a^	7.04 ± 0.64^b^	6.08 ± 0.48^b^	2.01 ± 0.44^d^	4.60 ± 0.52^c^
1692	1-Heptadecene	nd	nd	nd	7.56 ± 0.51^a^	0.70 ± 0.06^b^
	Subtotal	567.59 ± 72.94^a^	579.64 ± 45.95^a^	554.28 ± 60.27^a^	567.17 ± 46.77^a^	537.12 ± 28.02^a^
	*Other compounds*					
917	Diethyl disulfide	nd	2.93 ± 0.27^a^	3.11 ± 0.20^a^	nd	nd
991	2-Pentyl-furan	2.19 ± 0.38^a^	nd	nd	nd	nd
1029	Limonene	3.67 ± 0.69^a^	3.32 ± 0.43^a^	3.23 ± 0.21^a^	1.23 ± 0.26^b^	1.26 ± 0.12^b^
1515	2,4-Bis(1,1-dimethylethyl)-phenol	46.85 ± 5.33^a^	23.11 ± 2.82^b^	22.55 ± 1.99^b^	19.20 ± 1.22^b^	19.99 ± 1.89^b^
	Subtotal	52.71 ± 6.06^a^	29.36 ± 3.49^b^	28.89 ± 2.39^b^	20.43 ± 1.47^c^	21.26 ± 1.91^c^
	Total	1145.32 ± 116.21^b^	1125.33 ± 56.95^b^	1239.52 ± 102.70^b^	1157.22 ± 46.55^b^	2810.78 ± 142.56^a^

**Table 2:** OAVs of volatile compounds in yogurt fermented by *S. thermophilus* at different times.

Compound names	Odor descriptor	Odor threshold (mg·kg^−1^)	OAV				
			0 h	1.5 h	3.0 h	4.5 h	6.0 h
*Aldehydes*							
Benzaldehyde	Almond, burnt sugar^3^	0.35	3.8	5.3	5.8	9.2	10.5
Octanal	Fat, lemon, green^3^	0.0009	1608.2	1831.1	1661.1	842.6	1423.4
Benzeneacetaldehyde	Sweet, flora^24^	0.004	0.0	0.0	315.3	0.0	0.0
Nonanal	Sweet, floral, citrus, grass^3^	0.04	213.4	106.9	83.4	41.9	86.9
Decanal	Fatty^3^	0.03	78.3	39.9	38.0	42.5	0.0
(E)-2-Decenal	Fatty, waxy, green^24^	0.001	0.0	640.7	0.0	0.0	0.0
Undecanal	Fatty^3^	0.01	0.0	0.0	0.0	0.0	255.7
(E,E)-2,4-Decadienal	Fatty, green^24^	0.0001	22156.6	14623.5	17060.5	17810.2	22278.7
Dodecanal	Soapy, waxy, citrus^24^	0.0015	5836.5	3535.7	4192.9	615.0	0.0
*Ketones*							
2-Heptanone	Fresh cream^25^	0.14	386.6	404.1	466.1	437.0	513.1
2-Nonanone	Grass, fruity, floral^25^	0.08	808.4	816.7	994.0	760.5	833.9
2-Nonen-4-one	Fruity, floral^3^	0.0009	0.0	0.0	0.0	3391.7	2870.4
2-Decanone	Sweet, waxy^3^	0.08	56.5	42.3	44.2	0.0	0.0
2-Undecanone	Orange, grass, fresh^25^	0.08	1102.6	1054.7	1427.2	482.7	572.3
2-Dodecanone	Orange, grass, fresh^24^	0.08	24.8	28.3	34.8	0.0	0.0
*Acids*							
Hexanoic acid	Spicy, rancidity^25^	1.84	0.0	0.0	0.0	2.8	16.4
Octanoic acid	Chess, sweaty^25^	1.9	0.0	0.3	0.4	29.4	78.6
Decanoic acid	Rancid^3^	3	0.0	0.0	0.9	23.6	260.7
Undecanoic acid	Rancid^24^	10	0.0	0.0	0.0	1.0	1.6
Dodecanoic acid	Rancid^24^	10	1.1	0.2	0.6	6.7	56.4
Tridecanoic acid	Rancid^24^	10	0.0	0.0	0.0	0.0	0.6
Tetradecanoic acid	Rancid^24^	10	0.1	0.5	2.1	4.2	30.0
*Benzene derivatives*							
Ethylbenzene	Gasoline^24^	0.2	19.9	26.2	27.2	26.4	27.7
p-Xylene	Phenolic^24^	1	13.2	15.1	13.5	13.6	13.4
Styrene	Sweet, balsamic^24^	0.05	254.2	245.5	238.7	203.9	220.9
Naphthalene	Phenolic^24^	0.006	190.6	144.0	119.1	166.0	190.0
1-Methyl-naphthalene	Phenolic^24^	0.0075	465.1	266.7	278.4	301.4	0.0
*Other compounds*							
Diethyl disulfide	Cabbage, sulfide, gasoline^25^	0.01	0.0	293.0	310.7	0.0	0.0
2-Pentyl-furan	Green, fat^3^	0.006	364.9	0.0	0.0	0.0	0.0
Limonene	Orange, mint^25^	0.2	18.4	16.6	16.2	6.2	6.3

Odor description and threshold were provided in references [3, 24, 25].

## Data Availability

The data used to support the findings of this study are included within the article.
